# On-Demand Fabrication of Si/SiO_2_ Nanowire Arrays by Nanosphere Lithography and Subsequent Thermal Oxidation

**DOI:** 10.1186/s11671-017-1883-5

**Published:** 2017-02-09

**Authors:** Huaxiang Cao, Xinhua Li, Bukang Zhou, Tao Chen, Tongfei Shi, Jianqiang Zheng, Guangqiang Liu, Yuqi Wang

**Affiliations:** 1 Key Laboratory of Material Physics, Institute of Solid State Physics, Chinese Academy of Sciences, Hefei, 230031 People’s Republic of China; 2 University of Science and Technology of China, Hefei, 230026 People’s Republic of China

**Keywords:** Nanosphere lithography, Metal-assisted chemical etching (MACE), Nanowire arrays, Thermal oxidation

## Abstract

We demonstrate the fabrication of the large-area arrays of vertically aligned Si/SiO_2_ nanowires with full tunability of the geometry of the single nanowires by the metal-assisted chemical etching technique and the following thermal oxidation process. To fabricate the geometry controllable Si/SiO_2_ nanowire (NW) arrays, two critical issues relating with the size control of polystyrene reduction and oxide thickness evolution are investigated. Through analyzing the morphology evolutions of polystyrene particles, we give a quantitative description on the diameter variations of polystyrene particles with the etching time of plasma etching. Based on this, pure Si NW arrays with controllable geometry are generated. Then the oxide dynamic of Si NW is analyzed by the extended Deal-Grove model. By control, the initial Si NWs and the thermal oxidation time, the well-aligned Si/SiO_2_ composite NW arrays with controllable geometry are obtained.

## Background

Due to their unique chemical, optical, and electrical properties, Silicon nanowire (Si NW) structures are the important bases of the next generation of high efficiency and low-cost electronic devices [1–5]. In the past decades, intensive study has been paid on the fabrication of pure Si NW arrays with the ability to tune the wire density and dimensions [6–8]. Recently, more and more researchers are focusing on a composite nanomaterials, which consists of crystalline Si NW in the core and an amorphous SiO_2_ layer in the shell [9–11]. In many practical applications, the addition of a transparent dielectric nanoshell on the core Si NW has exhibited both optical and electrical benefits [12]. For example, in photovoltaic design, it has been presented that the nano-shelled Si NW can provide light absorption and external quantum efficiency over 100% under both transverse electric and magnetic incidences. Meanwhile, the SiO_2_ shell can serve as a passivation layer and assist in lowering the photocurrent loss due to surface carrier recombination [5, 13, 14]. In Lithium rechargeable batteries design, Si/SiO_2_ NW may offer a practical solution as new anode materials, where the unstoichiometric oxygen coordinations can provide an alternative electrochemical route, ensuing a marginal volume change with a potentially lower activation energy [9, 10, 15]. Obviously, these composite nanomaterials may offer a combined functionality contributed by these different components. But before this core-shell structure nanomaterial becomes available for mainstream applications in integrated devices, large-scale and deterministic assembly of Si/SiO_2_ NWs with rationally controlled geometries has to be realized.

By reference to the documents on the fabrication of pure Si NW arrays, one can find numerous methods that have been developed to fabricate Si nanostructure by using bottom-up or top-down approaches, such as vapor-liquid-solid (VLS) growth [16, 17], reactive ion etching [18], or electrochemical etching [19]. Among these methods, VLS growth is the most commonly used bottom-up method to produce Si NWs arrays, but the high temperature required for synthesis, the incorporation of metal impurities, the difficulties in controlling NWs diameter, lack of large-scale NWs placement technology hinder widespread application. Recently, the metal-assisted chemical etching (MACE) method has been demonstrated to produce ordered Si NW arrays [20], combined with monolayer nanosphere lithography (NSL) template technology [6, 21]. The advantages of this technique are manifold: the large availability of highly monodisperse particles allows precise control over the feature size, and the process is inherently parallel and scalable to large samples.

After Si NW arrays were fabricated, the following thermal oxidization technique was used to form SiO2 oxide layer on the surface of NWs. Compared with other SiO2 growth methods (i.e., plasma-enhanced chemical vapor deposition), the Si/SiO2 NWs prepared by thermal oxidation process have less surface defect density [14, 22] and no undesirable Si–OH and Si–H bonds [23, 24].

In view of the merits of the MACE and thermal oxidization technique, in this work, we developed a method to obtain the Si/SiO_2_ NWs with core-shell structure, which consists of the NWs’ fabrication by normal MACE technique and the following thermal oxidization of as-etched NW. Through quantitatively investigating the size reduction of polystyrene (PS) sphere and thermal oxidation dynamics in detail, the precise control over dimensions of the Si/SiO_2_ core-shell structure can be successfully realized. Based on this, large-scale core-shell Si/SiO_2_ NW arrays with tunable shell thicknesses and the inner diameter can be realized.

## Methods

The n-type Si (100) wafers (ρ~3 Ω∙cm) were cut into 1 × 1 cm pieces, degreased by ultrasonic cleaning in acetone, ethanol, and deionized water and subjected to boiling Piranha solution (3:1 (v/v) H_2_SO_4_/H_2_O_2_) for 1 h. Then, hexagonally close-packed PS colloidal sphere monolayer was synthesized by gas/liquid interface self-assembly on the silicon substrate. The PS sphere (*D*_*0*_ = 483 nm) solution (10 wt%) was purchased from Bangs Laboratories, Inc. (Fishers, IN, USA). After the colloidal sphere monolayer coating, the diameter of the PS spheres was reduced via plasma etching with an anisotropic Ar flow rate of 16.5 sccm, a pressure of 2.0 Pa, and a RF power of 200 W. Then, a Ti(2 nm)/Au(25 nm) bilayer film was sequentially evaporated on onto the Si substrate by electron beam deposition. Subsequently, the PS particle template was removed from the substrate by ultrasonication in toluene to form an ordered catalytic metal meshes. Then MACE were performed by immersing the Si wafer with the catalytic metal meshes in a mixture of etching solution (10:2:10, v/v/v, HF/H_2_O_2_/H_2_O) for several minutes at room temperature. After the formation of Si NW arrays by MACE, the diameter of the as-prepared Si NWs was reduced by the thermal oxidation process. Before putting the sample into the thermal oxidation furnace, the obtained Si NW arrays in the above step were rinsed with aqua regia to remove Au mesh. Then, Si NW arrays were transferred into a home-made thermal oxide tube furnace to the desired temperature at a rate of 50 °C min − 1 in an N_2_ atmosphere flowing at 100 sccm. When the desired temperature is stabilized, the feed gas was changed to pure O_2_ flowing at 100 sccm. Oxidation time was varied between 10 min and 3 h. Once the desired oxidation time was reached, the feed gas was changed back to N_2_, and the furnace was cooled down to room temperature at 10 °C min^−1^.

The morphology of the PS microspheres, the Ti/Au mesh, and the Si NW arrays were measured by the scanning electron microscope (SEM, Sirion 200 field emission scanning electron microscope). The crystallinity and the thicknesses of the core and shell layers of Si/SiO_2_ NWs were characterized by transmission electron microscopy (TEM) (JEOL JEM-2010). For TEM observation, the NWs were removed from the substrate with a scalpel and dispersed into a few droplets of methanol. Then, a TEM grid with a carbon film on a copper net was pulled through the solution.

## Results and Discussion

**Fig. 1:**
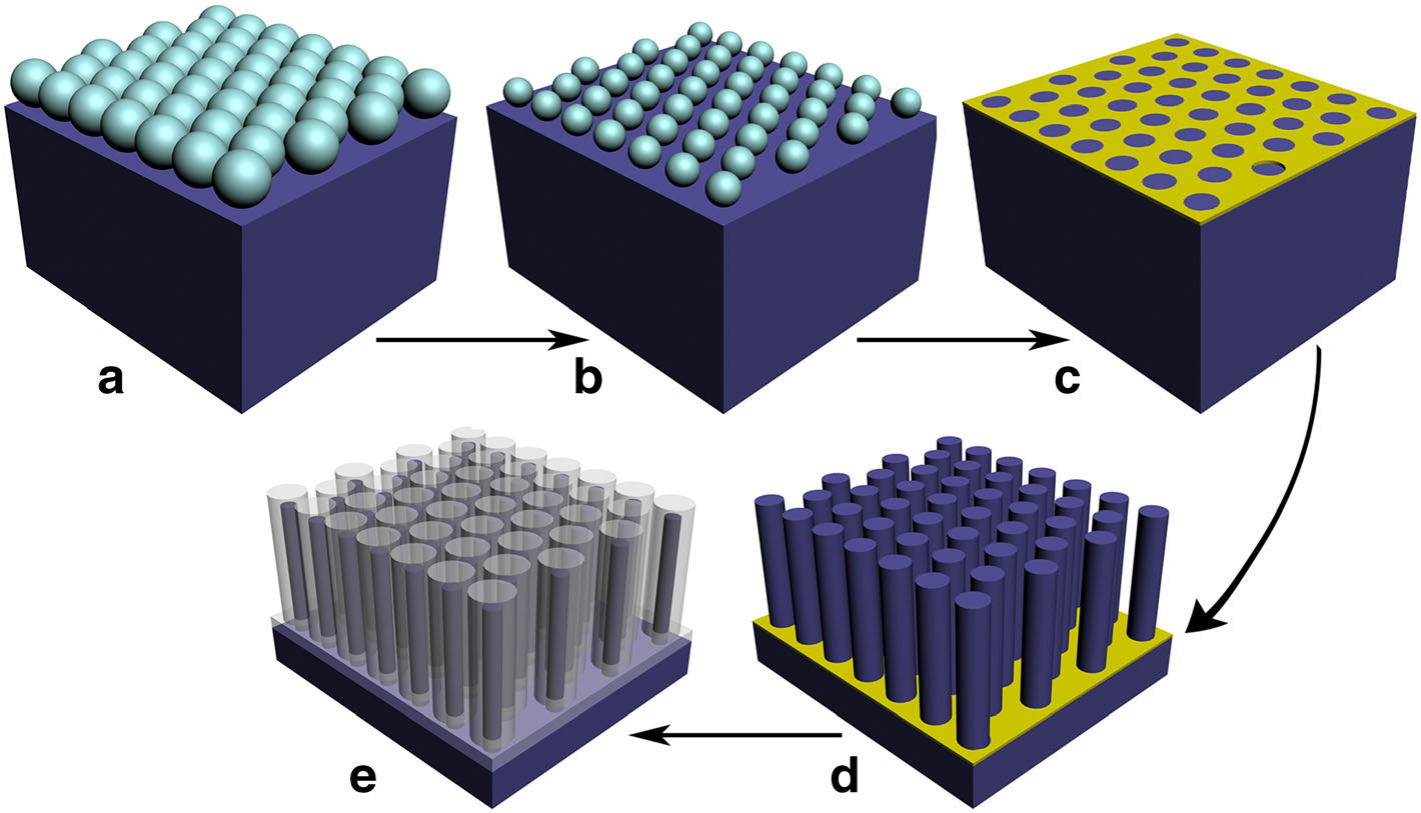
A schematic diagram of Si/SiO_2_ NW arrays fabrication. **a** A self-assembled colloidal monolayer was formed. **b** Non-close-packed colloidal monolayer was obtained after argon plasma treatment. **c** A Ti/Au film was evaporated on top, and the colloidal monolayer was peeled off by ultrasonication in toluene. **d** Si NW arrays etched via MACE using gold mesh as catalysts. **e** *Gold mesh* was dissolved in aqua regia and Si/SiO_2_ core-shell cylindrical structure was obtained by the following thermal oxidation process

Figure 1 shows the key fabrication steps to generate Si/SiO_2_ NW arrays. Before realizing the fabrication of Si/SiO_2_ NW arrays, it is necessary to prepare pure Si NW arrays with well-controlled geometry by MACE combined with NSL. From Fig. 1c, d, one can find that the NW cross-section is translated directly from the morphology of the reduced PS particles. Therefore, precise control over the diameter of PS particles during continued plasma etching reduction has become the main challenge in determining the diameter of Si NW.

**Fig. 2:**
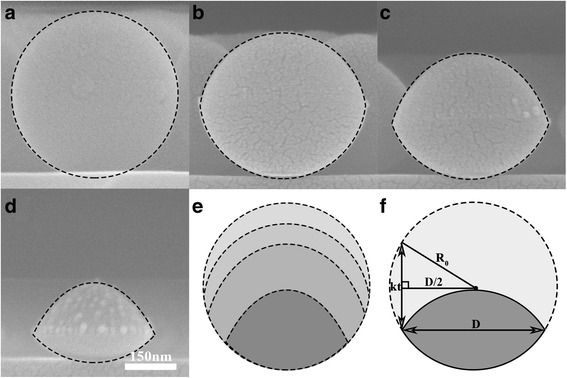
Cross-sectional SEM micrographs of PS particles after varying exposure durations. PS spheres with an initial diameter of 483 nm were etched for (**a**) 0, (**b**) 25, (**c**) 50, and (**d**) 100 s at 16.5 sccm Ar gas flows. The *black dashed elliptical* ones circling the etched particles assist to define the outlines of these etched spheres. The *outlines* of these etched particles are bottom-aligned in (**e**). **f** A schematic diagram of etching model

In order to control the diameter of PS particles effectively, we first investigate the morphology evolutions of PS particles after different exposure durations (0, 25, 50, and 100 s) to plasma etching (Fig. 2a–d). After the plasma treatment, the PS spheres are transformed from an isotropic spherical shape into a non-spherically morphology without affecting the original periodic and ordered arrangement of the particles. Most interestingly, the curvatures of bottom profiles of these PS particles after the plasma etching duration match well with that of untreated one, as shown in Fig. 2e. This implies few etching occurs at the lower part of the PS spheres. Hence, a near complete anisotropy etching of PS sphere can be obtained, which is facilitated by the parallel-plate electrode plasma reactor. Moreover, in this study, Argon with stable flow was used as the etching gas. This ensures only physical sputtering rather than chemical reactions occur at the top surface of PS spheres.

Based on the above analysis, the etching model was proposed to fit the diameter evolutions of the etched particles. As illustrated in Fig. 2f, we denote the radius of the PS particles as *R*_*0*_, the width of the resultant particle parallel to the substrate surface as the traverse diameter *D*. We further defined size reduction rate along the longitudinal direction as the etch rate *k*, as shown in Fig. 2f. We can approximate the relationship between traverse diameter *D* of reduced PS and argon plasma etching time *t* as:

1 $$ D=\sqrt{4{R_0}^2-{k}^2{t}^2} $$

Equation 1 describes a non-linear trend of PS sphere’s size shrinkage with plasma exposure time for fixed plasma parameters. In the previous studies, the linear relationship between the diameter of the particles and etching time are always reported. But this linear relationship is always observed under oxygen plasma treatments, in which isotropically shrinkages of the PS particles occur. In our study, subjected to anisotropy etching of the argon plasma, the PS particles exhibit a non-spherical shape even at short etching times. This causes the relations between colloid diameter and etching time deviate from line behavior. Chiseki et al. approximate the thinned PS particle diameter *D* and the oxygen etching time by a cosine equation [25]. But this non-linear relationship is based on empirical fitting. In their report, no clear physical mode was established. In this study, the relations between colloid diameter and etching time as presented in Eq. 1 are derived based on the model illustrated in Fig. 2f.

**Fig. 3:**
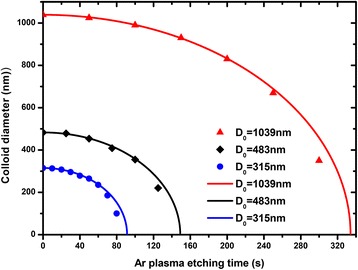
Reduction of the PS particle diameter vs etching time. PS spheres with various initial sphere diameter *D*_*0*_ (1039, 483, 315 nm) were treated with rf plasma at flow rates of 16.5 sccm Ar. The *symbols* are the experimental data and the *solid curves* is obtained from Eq. 1

To verify the feasibility of Eq. 1, the PS spheres with various initial diameter *D*_*0*_ (1039, 483, 315 nm) were treated with argon plasma for varying plasma exposure times. As shown in Fig. 3, red triangle symbols (1039 nm), black rhombus symbols (483 nm), and blue cycle symbols (315) correspond to experimental data. The solid curve is defined by Eq. 1 where *k* = 3.11, 3.24, 3.45 nm/s correspond to initial sphere diameter 1039 nm (red curve), 483 nm (blue curve), and 315 nm (blue curve). We find that Eq. 1 reproduces the experimental data well at short etching duration. As the particle diameter is further reduced to less than half of its initial value, the experimental points fall below the theoretical value calculated with Eq. 1. It should be noted that Eq. 1 is suitable for the complete anisotropy etching of nanosphere. After much longer exposure times (Fig. 2d), the lateral etching may occur at the side wall of PS particle, which causes the theoretical estimates calculated by Eq. 1 deviate from that of the experimental results. But before the diameters of the PS spheres are reduced by less than approximately half, Eq. 1 gives a precise description on the traverse diameter variations under the plasma treatment.

**Fig. 4:**
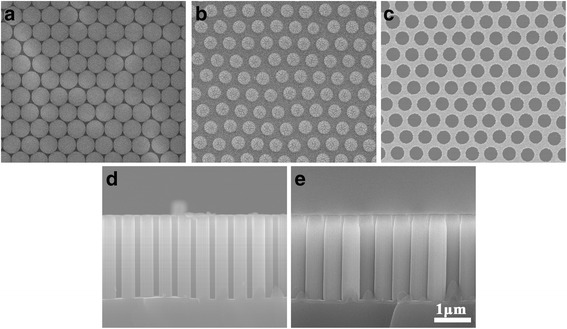
SEM images of Si/SiO_2_ NW arrays fabrication. **a** Plain view SEM morphology of self-assembled colloidal monolayer. **b** Plain view of the reduced PS particles treated by the anisotropic Ar plasma etching for 100 s. **c** Continuous Au mesh after the PS particles is removed. **d** Cross-sectional morphology of Si NW after etched by MACE. **e** Cross-sectional view of Si/SiO_2_ NW arrays after thermal oxidation at 1000°C for 1 h

The results in Fig. 3 confirm that the diameter of PS particles can be controlled by tuning the argon plasma etching time. To obtain Si NW arrays with diameter ~350 nm, one can select PS spheres with initial diameter 483 nm, and the corresponding plasma exposure time 102 s were calculated by Eq. 1. As shown in Fig. 4a, b, PS particles with diameter of ~351 nm were obtained after argon plasma exposure time 102 s. Subsequently, these reduced particles can serve as a lift-off masks and promote the formation of the conformal and continuous Au hole arrays with the same pitch but different size, as shown in Fig. 4b, c. After that, an MACE etching step is conducted in a mixture of deionized water, HF and H_2_O_2_. The continuous Au hole arrays catalyze the etching of silicon beneath it and promote the formation of the Si NW arrays. Figure 4d shows SEM images of the Si NW arrays, the formed edges on the sidewall of Si NWs were attribute to rough surface of etched PS particles. One can find that the cross-section of Si NW etched by the following MACE process matches well with the corresponding diameter and pitch of the catalyst nanohole. Therefore, starting with an ordered non-close-packed monolayer of microspheres, we can thus make use of this behavior described in Eq. 1 to generate Si NW with controllable geometry.

Based on the fabrication of the size-tunable Si NWs, we further prepare the Si/SiO_2_ NW arrays with core-shell structure by the thermal oxidization process. We next investigate the morphology evolution of Si NW arrays during the thermal oxidation process at 1000 °C. The Si NWs as etched by MACE technique have a uniform diameter (~350 nm) along the longitudinal direction (Fig. 4d). After oxidation, the oxidized NW exhibits a much larger diameter than its original Si NW, as shown in Fig. 4d, e. The diameter increase of the oxidized NW is mainly attributed to the atomic volume difference between SiO_2_ (45Å^3^) and Si (20Å^3^). By assuming the volume conservation during the thermal oxidation process, we can obtain the relationship between the diameter of resultant Si/SiO_2_ NW and initial Si NW as:

2 $$ \pi \left({b}^2-{a}^2\right)-\pi \left({b}_0^2-{a}_0^2\right)=\frac{V_{Si O2}}{V_{Si}}\pi \left({a}_0^2-{a}^2\right) $$

where *a* is the Si NW core radius, *b* is the total radius of the NW (NW core + SiO_2_ shell), *a*_*0*_ and *b*_*0*_ correspond to the initial values of *a* and *b* when the oxidation process is started.

$\frac{V_{Si O2}}{V_{Si}}=\frac{45}{20}$ is the relative SiO_2_/Si volume expansion ratio.

The diameter of NW before and after oxidation as shown in Fig. 4d, e basically obeys the relationship of Eq. 2. Thus, by Eq. 2, one can roughly estimate the dimensions of the Si/SiO_2_ core-shell structure.

**Fig. 5:**
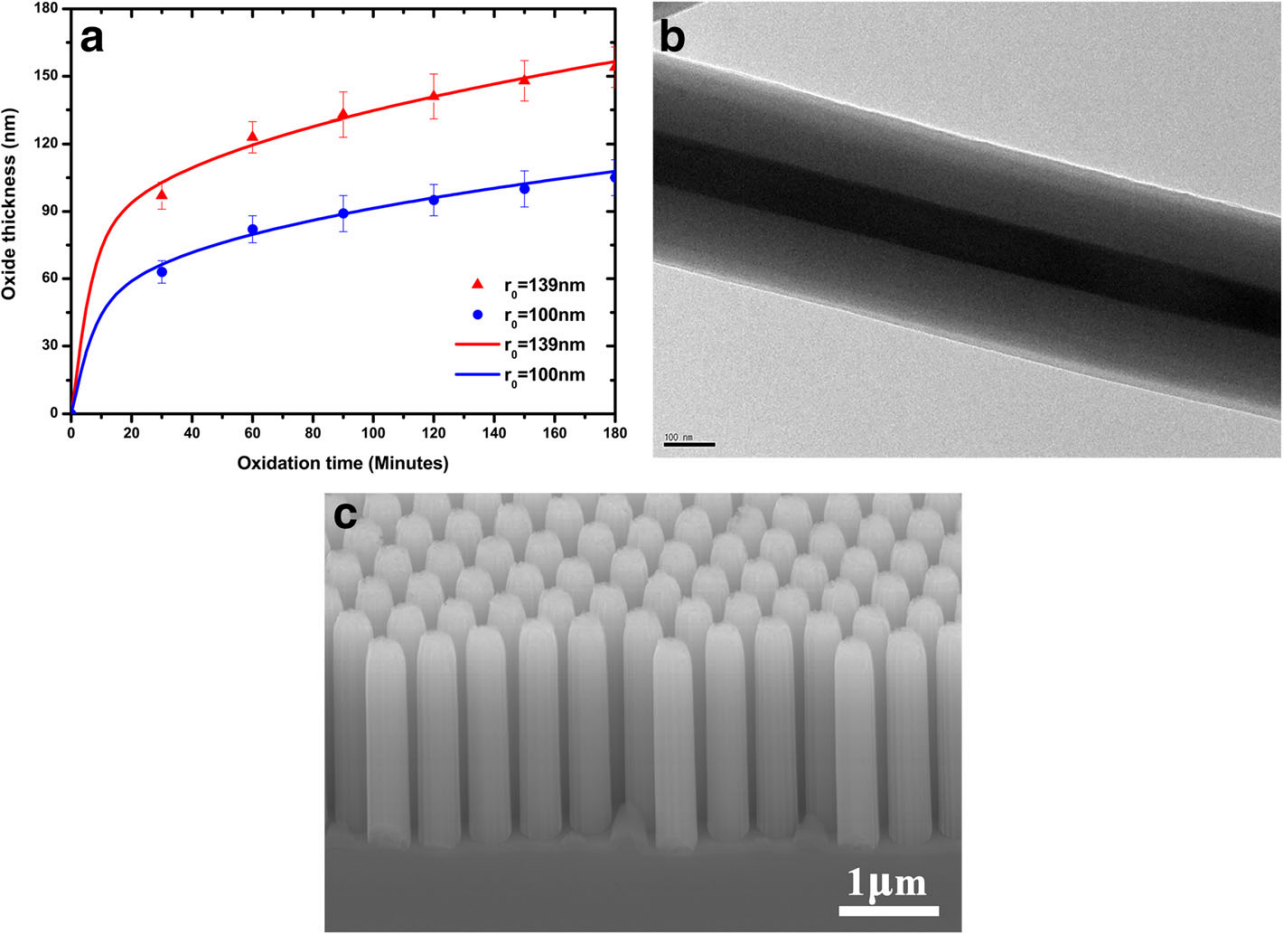
The evolution of oxide thicknesses of Si/SiO_2_ NW arrays during the thermal oxidation process. (**a**) Experimental data of oxide thickness as a function of time compared with theoretical results calculated by the extended Deal-Grove oxidation model with different starting radius *r*_*0*_ (139,100 nm) of Si NW under 1000° (*symbols* correspond to experimental points, *solid curves* correspond to the simulation results); (**b**) TEM image of individual Si/SiO_2_ composite NW and (**c**) SEM morphology of Si/SiO_2_ composite NW arrays after oxidation for 2.5 h at 1000° viewed from 45°

Another key technical issue to achieve the on-demand supply of the Si/SiO_2_ NW arrays is to predict the time required to reach the desired thickness of the thermal oxidation. This requires us to follow the oxidation kinetics of NW. Figure 5a shows the variation of the oxide thicknesses with the oxide time. It can be clearly observed that the oxidation rate declined quickly in 1 h. This phenomenon is consistent with previous reports on the retarded oxidation. The important factors underlying this behavior are the large curvature of the Si surface, which imposes compressive stress of the oxide in the radial direction due to volume expansion in the incorporation of O-atoms while forming SiO_2_. These dependences cannot be explained by the standard Deal-Grove oxidation model [26]. Furthermore, as the oxide thickness increases, the accumulated compressive stress in the radial direction can cause the decrease of the surface reaction rate and oxidant diffusivity at the Si/SiO_2_ interface. To precisely describe oxide dynamics of NW’s thermal oxidation, these factors must be taken into account. Considering, in this study, the oxidation time of Si NW was calculated by the extended Deal-Grove model [27, 28], which can be written in cylindrical coordinates as:

3 $$ \frac{\partial x}{\partial t}=\frac{1}{N}\frac{C^{*}}{1/{k}_s+\left(1/ h\right)\left( a/ b\right)+\left( a/ D\right) \log \left( b/ a\right)} $$

where *N* is the number of oxidants required to form a cubic unit of oxide, *a* is the Si NW core radius, *b* is the total radius of the NW (Si core + SiO_2_ shell), and *x* = *b*−*a* the oxide thickness. The physical parameters contained in this equation are the same in those used in the original Deal-Grove model: *N* is the number of oxidant molecules to incorporated into a unit volume of silicon oxide, *k*_*s*_ is the surface reaction rate constant at the SiO_2_/Si interface, *h* is the surface mass transfer constant of the oxidant, *D* is the diffusivity of oxidant in SiO_2_, and *C* ^*^ is the oxidant solubility in SiO_2_.

In this equation, both the reaction rate *k*_*s*_ at the Si/SiO_2_ interface and the diffusivity in the silicon oxide *D* are stress dependent. To take into account the effects of *P* (the tensile hydrostatic pressure inside the oxide volume) and *σ* (the compressive surface stress at the Si/SiO_2_ interface) on the oxidation parameter, we have used a “macroscopic” approach in which the activation energy of the various parameter is considered to vary as a result of the work applied by these forces on the oxide shell. As a consequence, *k*_*s*_, *D*, and *C* ^*^ and can be expressed as

4 $$ {k}_s={k}_{s,0}\cdot \exp \left(\frac{\sigma {V}_k}{ k T}\right), D={D}_0\cdot \exp \left(\frac{P{V}_d}{ k T}\right),{C}^{*}={C}_0^{*}\cdot \exp \left(\frac{P{V}_s}{ k T}\right) $$

with *k*_*s,0*_, *D*_*0*_, and *C*_0_^*^ are the the values of the coefficients in the case of planar oxidation while *V*_k_, *V*_d_, *V*_s_ are empirical parameters corresponding to the activation volumes.

*P* and *σ* as a function of *a* and *b* can be written as:

5 $$ P=2\eta \frac{\beta}{b^2},\sigma =-2\eta \beta \left(\frac{1}{a^2}-\frac{1}{b^2}\right) $$

where the function *β* is defined as $\beta = a\frac{dx}{dt}$.

Meanwhile, the dependence of viscosity non-linear on shearing stresses is described as:

6 $$ \eta ={\eta}_0(T)\left({\sigma}_s{V}_o/ kT\right)/ \sinh \left({\sigma}_s{V}_o/ kT\right) $$

where *η*_0_ denotes the low stress viscosity, *σ*_s_ is the shear-stress in oxide, and *V*_o_ is fitting parameters.

The parameters *η*_*0*_, *V*_*0*_, and others are obtained experimentally and presented in Table 1 [29, 30].

**Table 1:** The parameters used in the oxidation time calculation

Parameter	Description	Value
alpha(Pa^−1^)	Empirical parameter	1.5 × 10^−8^
*h*(m/s)	Surface mass transfer constant of oxidant	2.78 × 10^−2^
*N*(m^3^)	The number of oxidant molecules	2.25 × 10^28^
*V*_s_(m^3^)	Empirical parameters	2 × 10^−29^
*V*_d_(m^3^)	Empirical parameters	7.5 × 10^−29^
*V*_k_(m^3^)	Empirical parameters	2.5 × 10^−29^
*V*_0_(m^3^)	Fitting parameters	3.4 × 10^−28^
*k*_s_(m/s)	Surface reaction rate constant	5 × 10^−5^
*D*_0_(m^2^/s)	Diffusivity of oxidant	7.25 × 10^−14^
*C*_0_*(m^−3^)	Oxidant solubility	5.5 × 10^22^
*η*_0_ (Pa · s)	Oxide viscosity	7.6 × 10^15^

The oxidation process can be well-described (Fig. 5a) by solving Eqs. 3–6. The agreement between the experimental data (symbols) and the model results (solid curve) proves the validity of the model assumptions. Besides giving the useful insight on the mechanisms governing the PS size reduction and NW oxidation, these results also provide a powerful method for fabricating Si/SiO_2_ NW arrays with controllable geometries. For example, to obtain a core-shell Si/SiO_2_ NW with core diameter of 100 nm and shell thickness of 150 nm, one can select a starting NW diameter of 277 nm by solving the Eq. 2. If the initial diameter of the PS is 483 nm, the exposure durations to the argon plasmas were chosen as 122 s to obtain the resultant PS particle with diameter ~278 nm (by Eq. 1). Based on the non-close-packed PS template, the well-aligned Si NW arrays with the desired diameter can be obtained by the MACE technique. Subsequently, to obtain Si/SiO_2_ NW arrays with a shell thickness of 150 nm, the oxidation time of ~2.5 h can be obtained by the extended Deal-Grove oxidation model. Figure 5b shows the TEM images of the resulting core-shell structure of the Si/SiO_2_ NWs. The inner part shows the silicon core which is 96 nm in diameter. The outer shell consists of silicon dioxide and its thickness is 149 nm. The TEM results prove the validity of the model. The good agreements of the experimental data and the model results also suggest that, through selecting the initial diameter and oxidation time of Si NW, the Si/SiO_2_ NW arrays can be fabricated easily in terms of experimental requirements. Finally, the Si/SiO_2_ NW arrays were qualitatively investigated by SEM. Figure 5c shows low-magnification SEM images of the Si/SiO_2_ NW arrays that were fabricated by MACE technique and the following thermal oxidization. The well-aligned and relatively smooth Si/SiO_2_ composite NW arrays with good periodicity prove that large-scale Si/SiO_2_ NW arrays with controllable geometry were realized.

## Conclusions

In conclusion, large-scale and deterministic assembly of Si/SiO_2_ NW arrays are realized by the MACE combined with the following thermal oxidization process. To realize the on-demand fabrication of this composite structure, two critical issues determining the geometries of the final Si/SiO_2_ NW arrays are investigated. We first proposed a non-linear equation to estimate the diameter variations of PS particles under the plasma treatment. By the quantitative character described in Eq. 1, pure Si NWs with controllable geometry are fabricated. Then, the evolution of oxide thicknesses of Si NW arrays during the thermal oxidation process is investigated in detail. By the extended Deal-Grove model, the initial Si NW and the thermal oxidation time required can be estimated. Based on this, the well-aligned Si/SiO_2_ composite NW arrays with controllable geometry are obtained. This Si/SiO_2_ composite NW arrays have great potential application in the next generation of high efficiency and low-cost photo-electrical devices.

## Abbreviations

MACE: Metal-assisted chemical etching
NSL: Nanosphere lithography
PS: Polystyrene
SEM: Scanning electron microscope
Si NW: Silicon nanowire
TEM: Transmission electron microscopy
VLS: Vapor-liquid-solid

## References

[1] Chan CK, Peng H, Liu G, McIlwrath K, Zhang XF, Huggins RA, Cui Y (2008). High-performance lithium battery anodes using silicon nanowires. Nat Nano.

[2] Brongersma ML, Cui Y, Fan S (2014). Light management for photovoltaics using high-index nanostructures. Nat Mater.

[3] Yan H, Choe HS, Nam S, Hu Y, Das S, Klemic JF, Ellenbogen JC, Lieber CM (2011). Programmable nanowire circuits for nanoprocessors. Nature.

[4] Luo L, Yang H, Yan P, Travis JJ, Lee Y, Liu N, Molina Piper D, Lee S-H, Zhao P, George SM (2015). Surface-coating regulated lithiation kinetics and degradation in Silicon nanowires for lithium ion battery. ACS Nano.

[5] Yu P, Wu J, Liu S, Xiong J, Jagadish C, Wang ZM (2016). Design and fabrication of silicon nanowires towards efficient solar cells. Nano Today.

[6] Huang Z, Fang H, Zhu J (2007). Fabrication of silicon nanowire arrays with controlled diameter, length, and density. Adv Mater.

[7] Liu L, Peng K-Q, Hu Y, Wu X-L, Lee S-T (2014). Fabrication of silicon nanowire arrays by macroscopic galvanic cell-driven metal catalyzed electroless etching in aerated HF solution. Adv Mater.

[8] Chang S-W, Chuang VP, Boles ST, Ross CA, Thompson CV (2009). Densely packed arrays of ultra-high-aspect-ratio silicon nanowires fabricated using block-copolymer lithography and metal-assisted etching. Adv Funct Mater.

[9] Sim S, Oh P, Park S, Cho J (2013). Critical thickness of SiO2 coating layer on core@shell bulk@nanowire Si anode materials for Li-Ion batteries. Adv Mater.

[10] Kim S, Park J-S, Chang KJ (2012). Stability and segregation of B and P dopants in Si/SiO2 core–shell nanowires. Nano Lett.

[11] Jinyou X, Pengfei G, Zhijun Z, Yang L, Hailong Y, Yongsong L (2016). Eu-doped Si-SiO 2 core–shell nanowires for Si-compatible red emission. Nanotechnology.

[12] Seo K, Yu YJ, Duane P, Zhu W, Park H, Wober M, Crozier KB (2013). Si microwire solar cells: improved efficiency with a conformal SiO2 layer. ACS Nano.

[13] Mallorquí AD, Alarcón-Lladó E, Mundet IC, Kiani A, Demaurex B, De Wolf S, Menzel A, Zacharias M, Fontcuberta i Morral A (2015). Field-effect passivation on silicon nanowire solar cells. Nano Res.

[14] Oh J, Yuan HC, Branz HM (2012). An 18.2%-efficient black-silicon solar cell achieved through control of carrier recombination in nanostructures. Nat Nanotechnol.

[15] Zhang C, Gu L, Kaskhedikar N, Cui G, Maier J (2013). Preparation of silicon@silicon oxide core–shell nanowires from a silica precursor toward a high energy density Li-Ion battery anode. ACS Appl Mater Interfaces.

[16] Wu Y, Yang P (2001). Direct observation of vapor–liquid–solid nanowire growth. J Am Chem Soc.

[17] Kodambaka S, Tersoff J, Reuter MC, Ross FM (2006). Diameter-independent kinetics in the vapor-liquid-solid growth of Si nanowires. Phys Rev Lett.

[18] Fu YQ, Colli A, Fasoli A, Luo JK, Flewitt AJ, Ferrari AC, Milne WI (2009). Deep reactive ion etching as a tool for nanostructure fabrication. J Vac Sci Technol B: Microelectron Nanometer Struct–Process Meas Phenom.

[19] Juhasz R, Elfström N, Linnros J (2005). Controlled fabrication of silicon nanowires by electron beam lithography and electrochemical size reduction. Nano Lett.

[20] Peng KQ, Yan YJ, Gao SP, Zhu J (2002). Synthesis of large-area silicon nanowire arrays via self-assembling nanoelectrochemistry. Adv Mater.

[21] Yeom J, Ratchford D, Field CR, Brintlinger TH, Pehrsson PE (2014). Decoupling diameter and pitch in silicon nanowire arrays made by metal-assisted chemical etching. Adv Funct Mater.

[22] Lee Y, Oh W, Dao VA, Hussain SQ, Yi J (2012). Ultrathin oxide passivation layer by rapid thermal oxidation for the silicon heterojunction solar cell applications. Int J Photoenergy.

[23] Schliwinski HJ, Schnakenberg U, Windbracke W, Neff H, Lange P (1992). Thermal annealing effects on the mechanical properties of plasma‐enhanced chemical vapor deposited silicon oxide films. J Electrochem Soc.

[24] Sassella A, Borghesi A, Corni F, Monelli A, Ottaviani G, Tonini R, Pivac B, Bacchetta M, Zanotti L (1997). Infrared study of Si-rich silicon oxide films deposited by plasma-enhanced chemical vapor deposition. J Vac Sci Technol A.

[25] Haginoya C, Ishibashi M, Koike K (1997). Nanostructure array fabrication with a size-controllable natural lithography. Appl Phys Lett.

[26] Deal BE, Grove AS (1965). General relationship for the thermal oxidation of silicon. J Appl Phys.

[27] Egorkin AV, Kalinin SV (2014). Two-dimensional modeling the process of thermal oxidation of non-planar silicon structures in CMOS-circuits’ isolation. 2014 15th International Conference of Young Specialists on Micro/Nanotechnologies and Electron Devices (EDM): 2014.

[28] Kao D-B, McVittie JP, Nix WD, Saraswat KC (1988). Two-dimensional thermal oxidation of silicon. II. Modeling stress effects in wet oxides. IEEE Trans Electron Devices.

[29] Kurstjens R, Vos I, Dross F, Poortmans J, Mertens R (2012). Thermal oxidation of a densely packed array of vertical si nanowires. J Electrochem Soc.

[30] Coffin H, Bonafos C, Schamm S, Cherkashin N, Assayag GB, Claverie A, Respaud M, Dimitrakis P, Normand P (2006). Oxidation of Si nanocrystals fabricated by ultralow-energy ion implantation in thin SiO2 layers. J Appl Phys.

